# Adaptive Strategies and Underlying Response Mechanisms of Ciliates to Salinity Change with Note on Fluctuation Properties

**DOI:** 10.3390/microorganisms12101957

**Published:** 2024-09-27

**Authors:** Fenfen Li, Jing Yang, Jiqiu Li, Xiaofeng Lin

**Affiliations:** 1 Key Laboratory of Ministry of Education for Coastal and Wetland Ecosystems, Fujian Provincial Key Laboratory for Coastal Ecology and Environmental Studies, College of the Environment and Ecology, Xiamen University, Xiamen 361102, China; lifenfen1175@163.com; 2 State Key Laboratory of Marine Environmental Science, Xiamen University, Xiamen 361102, China; 3 College of Life Science, South China Normal University, Guangzhou 510631, China; yang_jing4567@126.com

**Keywords:** adaptive strategy, ciliated protozoa, fluctuation property, response mechanism, salinity change

## Abstract

The adaptability of marine organisms to changes in salinity has been a significant research area under global climate change. However, the underlying mechanisms of this adaptability remain a debated subject. We hypothesize that neglecting salinity fluctuation properties is a key contributing factor to the controversy. The ciliate *Euplotes vannus* was used as the model organism, with two salinity fluctuation period sets: acute (24 h) and chronic (336 h). We examined its population growth dynamics and energy metabolism parameters following exposure to salinity levels from 15‰ to 50‰. The carrying capacity (*K*) decreased with increasing salinity under both acute and chronic stresses. The intrinsic growth rate (*r*) decreased with increasing salinity under acute stress. Under chronic stress, the *r* initially increased with stress intensity before decreasing when salinity exceeded 40‰. Overall, glycogen and lipid content decreased with stress increasing and were significantly higher in the acute stress set compared to the chronic one. Both hypotonic and hypertonic stresses enhanced the activities of metabolic enzymes. A trade-off between survival and reproduction was observed, prioritizing survival under acute stress. Under chronic stress, the weight on reproduction increased in significance. In conclusion, the tested ciliates adopted an *r*-strategy in response to salinity stress. The trade-off between reproduction and survival is a significant biological response mechanism varying with salinity fluctuation properties.

## 1. Introduction

In the context of global climate change, the adaptive strategies and underlying response mechanisms of organisms to environmental changes have become a focal point in ecological research [1,2,3]. As a critical environmental factor in the marine ecosystem, salinity fluctuations and their ecological implications have long been a subject of scientific inquiry [4,5,6]. Extensive research has shown that salinity gradients significantly influence marine biodiversity and species distribution, from microbes to vertebrates [7,8,9]. Nevertheless, the precise mechanisms underlying the ecological impacts of salinity fluctuations remain elusive. Importantly, a comprehensive understanding of the biological response mechanisms and adaptability of various biological taxa to salinity changes is essential for unraveling these mechanisms. Unfortunately, considerable disagreement persists regarding the conclusions drawn from studies within this domain. For instance, in fish (*Lutjanus campechanus*) that were reared for 60 days at 8‰ after being moved from an in situ 32‰, the survival rate remained unaffected, while the growth rate was significantly reduced [4,10]. Moreover, shrimp (*Litopenaeus vannamei*) cultured at 2‰ for 40 days exhibited significantly reduced survival and growth rates compared to those grown in situ at 30‰ [11]. In contrast, wild rabbitfish (*Siganus guttatus*) cultured at 10‰ for 6 weeks showed higher survival rates, specific growth rates, and final weight compared to those cultured at 30‰ [12]. Furthermore, a consensus was formed that appropriate hypotonic treatment of seawater fish or high permeability treatment of freshwater fish can improve their development and growth rates [13]. Conversely, hypertonic treatments with increased salinity were shown to have inconsistent effects on aquatic organisms. For example, a significant reduction in reproductive performance and survival rates was observed in freshwater zooplankton exposed to high salt concentrations (approximately 19.7‰) over a 21-day life-history experiment [14]. Similarly, a 14-day exposure experiment revealed that both *Moina macrocopa* and *Daphnia magna* experienced significant reductions in survival time, growth rate, and carrying capacity when salinity increased from fresh water to 0.10 M salt water [15]. However, evidence suggests that appropriate hypertonic treatment can significantly enhance zooplankton performance. For instance, the growth rate of body length increased in *Moina affinis* as salinity increased from fresh water to 5‰ of salt water [16]. These studies indicate that the biological responses of the tested organisms and the ecological consequences varied with the properties of salinity fluctuations, such as fluctuation amplitude, direction of change (hypertonic or hypotonic stress), and duration of stress (acute and chronic exposure). In natural conditions, the salinity fluctuations of coastal ecosystems exhibit these properties due to ocean tidal flow, rainfall, evaporation, river freshwater input, and ice melting [17,18,19]. Consequently, it is crucial to account for the properties of salinity fluctuations in natural marine ecosystems, as this is likely a pivotal approach to reconcile disparities between different studies, particularly those conducted in laboratories and the field [20]. The following questions arise: Do the properties of salinity fluctuations influence the adaptive strategies of marine microorganisms? How will the underlying biological response mechanisms change? Answering these questions necessitates comprehensive research under a unified experimental system [21].

In terms of adaptive strategies, organisms have evolved two strategies to cope with environmental stress at the population level: *K*-strategy and *r*-strategy [11,22]. Briefly, the *r*-strategy involves producing larger clutches of smaller offspring, whereas the *K-*strategy is characterized by producing fewer, larger offspring [23]. Notably, the choice of adaptive strategies by organisms is influenced by both genetic factors and the characteristics of the environmental factors [22,24]. Therefore, this poses significant challenges in understanding the biological mechanisms that underpin adaptive strategies. In terms of biological responses, the level of biological organization dictates its role in indicating ecological significances and revealing response mechanisms. Generally, the lower the biological level, the more sensitive the biological responding, and the more important the roles in revealing the biological responding mechanisms [25]. Nevertheless, it is undeniable that the relationship between these levels is too complex and challenging to elucidate. A major challenge is to extrapolate responses at lower biological levels to those observed at higher levels [26]. A common method involves creating integrative indices based on multiple parameters to infer ecological effects at higher biological levels [27], such as the Integrative Biological Response [28]. Nevertheless, this approach also poses a complex challenge in quantifying the importance of each parameter within the adopted set [25]. Fortunately, energy metabolism parameters are increasingly acknowledged as a universal “currency” that links responses at different biological levels [29]. For example, the energy content (including lipid, glycogen, and protein) and the activity of metabolic enzymes (e.g., LDH) have been widely utilized in the assessment of ecological effects and the interpretation of the underlying mechanisms [29,30]. Nonetheless, there remains a knowledge gap regarding the adaptive mechanisms of aquatic animals to salinity stress, particularly in terms of energy use and supply [31].

Species-specificity responses are also a significant factor contributing to the variability of results in ecological research [32]. Considering the species-specificity response of organisms to salinity fluctuations, it is crucial to investigate the ecological effects of salinity fluctuation across a wider array of taxa [33]. Ciliated protozoa, as unicellular eukaryotes, are integral to marine microecosystems and are instrumental in material cycling and energy flow [34,35]. Moreover, ciliated protozoa possess several distinctive biological traits, including high species diversity, broad distribution, rapid reproduction, facile culture, and sensitivity to environmental shifts, which endow them with significant advantages for scientific study [36]. For example, ciliated protozoa are acknowledged as ideal indicator species in environmental science [37]. Moreover, they have been extensively employed as model organisms in life sciences and related disciplines [36]. Unfortunately, as far as we are aware, there is a dearth of research examining the ecological effects of salinity fluctuations on ciliated protozoa.

Given the research context outlined above, we propose the following hypothesis: Ciliated protozoa are expected to employ distinct adaptive strategies in response to salinity fluctuations, and these strategies will be underpinned by varying response mechanisms. To test this hypothesis, we chose *Euplotes vannus*, a euryhaline ciliate with 30‰ In-situ environmental salinity, as a model organism. We then assessed the population growth dynamics and energy metabolism parameters of *E. vannus* exposed to salt solutions with varying concentrations (15‰, 30‰, 35‰, 40‰, 45‰, and 50‰) for two different durations (24 h and 336 h) each. Based on these observations, we analyzed both the adaptive strategies and the underlying response mechanisms, highlighting the salinity fluctuation properties, such as fluctuation amplitude (salt concentration), direction of change (hypotonic or hypertonic stress), and duration of stress (acute and chronic exposure). Specifically, we sought to answer the following questions: (1) How do the population growth dynamics of the tested ciliates respond to salinity fluctuation properties? (2) How do the energy metabolism parameters adjust in response to these properties? (3) What is the correlation between these response parameters across different biological levels? To the best of our knowledge, this is the first study to investigate salinity adaptive strategies and their underlying mechanisms in ciliated protozoa. Specifically, this study has the following characteristics: from the perspective of salinity fluctuation properties, using energy parameters as evaluation indicators, and completing all tests in a unified experimental system. Therefore, this finding will not only fill a gap in the research field of ciliate biology but also provide a new perspective for the study the ecological effects of salinity change.

## 2. Materials and Methods

### 2.1. Organisms and Cultivation

*Euplotes vannus*, a ciliate, was employed as the test organism in this study, sourced from the Protozoology Ocean University of China, Qingdao, China. The species was collected from the coast with an in situ salinity of 30‰ [38]. Throughout the conservation period, the colonial cultures were maintained at the same salinity as in the collection environment, at 25 ± 1 °C, with rice grains to enrich natural bacteria as food for the ciliate.

### 2.2. Preparation of a Gradient-Concentration Salt Solution

For the salinity stress experiment, a series of concentration gradients (15‰, 30‰, 35‰, 40‰, 45‰, and 50‰) was established by diluting or concentrating Oshima’s artificial seawater (Oshima’s ASW, salinity 30‰). Oshima’s artificial seawater (Oshima’s ASW, salinity 30‰) was prepared by dissolving NaCl (28 g), KCl (0.8 g), MgCl_2_·6H_2_O (5 g), and CaCl_2_·H_2_O (1.2 g) in 1 L of distilled water (Watson, Hong Kong). The solution was then adjusted to pH 8.2 using 0.5 M NaOH and filtered through a 0.22 μm polycarbonate membrane (Millipore, Billerica, MA, USA) to remove undissolved salt particles. *E. vannus* was cultured in the aforementioned gradient concentrations of salinity solution, supplemented with 0.55 g/L protozoan pellets (Carolina Biological Supply, Burlington, NC, USA). These pellets were crushed, boiled for 30 min, and then cooled before being inoculated with the bacterium *Idiomarina* sp. *DYB* at a concentration of 10^8^ CFU/L. *Idiomarina* sp. *DYB*, a euryhaline bacterium, was used as food for *E. vannus* [20,39].

### 2.3. Experimental Design

To investigate the effect of salinity fluctuation on ciliates, *E. vannus* cells were cultured in salinity solutions across a gradient, with culture conditions set at 25 ± 1 °C. For the control (at 30‰, the in situ salinity), inoculation into 15‰ solution was considered as hypotonic stress, while inoculation into 35‰, 45‰, and 50‰ was classified as hypertonic stress. Additionally, to assess the effect of stress duration, acute and chronic experiments were designed, extending the duration of the previously described experiments.

In acute exposure experiments, aliquots of *E. vannus* in the logarithmic phase were inoculated into 50 mL salinity solutions, each with the aforementioned gradient salinity concentrations, and cultured for 24 h. The chronic exposure experiment extended the incubation to 336 h. To ensure adequate cell populations for subsequent analyses, at least five replicates were established for each salinity treatment, and each container contained enough cells, provided that the cell abundance was lower than the environmental capacity in this culture (as determined by pre-experiments). During the chronic exposure experiments, half of the culture medium was replaced by fresh medium at 24 h intervals to reduce the accumulation of metabolite toxins [40]. At the end of the stress experiments, ciliates were collected to measure various parameters, such as population growth dynamic, metabolic enzyme activity, glycogen content, and gene expression levels.

### 2.4. Determination of the Population Growth Dynamics

Dynamics of ciliate population growth were determined in a standard 6-well culture plate, as described in [25]. In this experiment, *E. vannus* that had been exposed to the above specified salinity stress was inoculated into salt solutions at the same concentrations used in the stress experiments. Each solution was allocated to triplicate wells, with each well inoculated with fifty *E. vannus* cells in a 6 mL solution at the start. Every 24 h, the cell count per well was recorded, and half of the medium was replaced with fresh medium. The experiment was terminated after the cell population reached the stationary phase.

In elucidating population growth dynamics, logistic growth curves were iteratively fitted to *Euplotes vannus* cell counts using the Marquardt–Levenberg least squares algorithm [40]. The parameters of the logistic growth curves were calculated using the following equations in SigmaPlot 14.0 (Systat Software, Inc., San Jose, CA, USA):

(1) $$N_{t}=\frac{K}{1+e^{-rt}\left[\left(\frac{K}{N_{0}}\right)-1\right]}$$

where *N_t_* is the population size at a given time point, *N_0_* is the population size at time zero, and *t* is culture time. The per capita growth rate *r* (24 h^−1^) and carrying capacity *K* (ind. mL^−1^) were obtained from the logistic growth curve along with standard error estimates.

### 2.5. Measurement of Enzyme Activity and Energy Storage Substance

Following the stress experiments, ciliates from each repetition were separately harvested by centrifugation at 6000 rpm and 25 °C for 10 min. The samples were employed to measure enzyme activity and other physiological parameters, including the activities of lactate dehydrogenase (LDH), malate dehydrogenase (MDH), and pyruvate kinase (PK), as well as glycogen and lipid content. Lactate dehydrogenase (LDH) catalyzes the reversible reaction of pyruvic acid into lactate. In this study, pyruvic acid reacts with 2,4-dinitrophenylhydrazine to form a brownish-red alkaline solution, which is pyruvate dinitrophenylhydrazone and can be quantified by microplate reading at 450 nm [41]. One unit of LDH activity is defined as a gram of protein reacting with the substrate at 37 °C for 15 min, and 1 µmol of pyruvate is produced in the reaction system. Malate dehydrogenase (MDH) catalyzes an oxidation-reduction reaction, leading to a decrease in absorbance at 340 nm. The enzyme activity is determined by measuring the change in absorbance per minute [42]. One unit of MDH activity is defined as the catalysis of 1 µmol of substrate to product per minute per milligram of tissue protein in this reaction system. Pyruvate kinase (PK) catalyzes phosphoenolpyruvate and ADP to generate ATP and pyruvate. Lactate dehydrogenase further catalyzes NADH and pyruvate to produce lactic acid and NAD^+^ [43]. One unit of PK activity is defined as the conversion of 1 µmol of PEP to pyruvate per minute per gram of tissue protein. Glycogen content was quantified using the anthrone method [44]. Glycogen was extracted using a strong alkaline extraction solution, and its content was measured using an anthranone chromogenic reagent at 620 nm under strong acidic conditions. Enzyme activities were measured using commercially available kits (Nanjing Jiancheng Bioengineering Institute, Nanjing, China) and a glycogen content kit (Solarbio Science and Technology Co., Beijing, China), following the manufacturers’ instructions. The measurement procedure followed the instructions provided by the manufacturer. Lipid extraction was optimized based on the methods [45]. The determination of lipid content was conducted following the method described in the literature [46]. The total protein content in each sample was quantified using the bicinchoninic acid (BCA) method [47], and these values were used to normalize enzyme activities (LDH, MDH, and PK) and the glycogen and lipid content. All experiments were conducted in triplicate. After conversion, enzyme activities were ultimately expressed as units per gram of tissue protein from crude enzyme. All protein content and physiological parameter measurements were performed using a microplate reader (BioTek Epoch 2, Winooski, VT, USA).

### 2.6. Gene Expression Levels of Energy Metabolism Enzymes

To elucidate the response mechanisms of the ciliates to salinity changes, there were six treatments in the gene expression experiment; that is, acute or chronic exposure experiments × three salt concentrations (15‰, 30‰, and 50‰). These three concentrations were selected for gene expression assays based on their effects on population growth traits and covering the approximate concentration range for growth experiments. The gene expression levels of metabolic enzymes, including malate dehydrogenase (*Ev*MDH) (GenBank accession no. MK617186), pyruvate kinase (*Ev*PK) (GenBank accession no. MK617187), and snf1-related protein kinase (*Ev*SnRK) (GenBank accession no. MK617188), were quantified in *E. vannus*. Details of the selection of internal control genes can be found in the references [48]. All reactions were performed in triplicate in 96-well plates, and relative gene expression levels were calculated using the 2^−ΔΔCT^ method [49]. Details of sample collection, total RNA extraction, gene cloning, cDNA synthesis, and real-time quantitative PCR (qPCR) are provided in the Supplementary Materials Text S1.

### 2.7. Statistical Analyses

Statistical analyses were performed using IBM SPSS Statistics software (version 22.0 for Windows). All statistical tests were conducted with a significance level of *p* < 0.05. Results are presented as means ± S.E. (standard error of the means). Graphs were created using GraphPad Prism version 8.0.2 (GraphPad Software, Inc., San Diego, CA, USA). Initially, the raw data were tested for normality of distribution (Shapiro–Wilk test) and homogeneity of variance (Levene test). If the data distribution adequately satisfied the assumptions of normality and homogeneity of variance, one-way ANOVA was employed to compare multiple treatments, and a *t*-test was used for paired samples with only two treatments. If significant overall differences were found among means, the least significant difference (LSD) test was used to compare specific means. Repeated measures analysis of variance (*r*-ANOVA) was conducted to evaluate the significant effects of salinity treatment, exposure duration, and treatment × duration interaction, where salinity was used as the between-subjects factor, and time was used as the within-subjects factor. If the assumptions of ANOVA or *t*-test were not met, nonparametric tests such as the Kruskal–Wallis test (for more than two treatments) or Kolmogorov–Smirnov (for two treatments) test were used to compare means. To analyze correlations between variables, separate Pearson correlation analyses were conducted for each set of variables measured across different experiments.

## 3. Results

### 3.1. Effects on the Logistic Growth Dynamics of Euplotes Vannus

**Figure 1:**
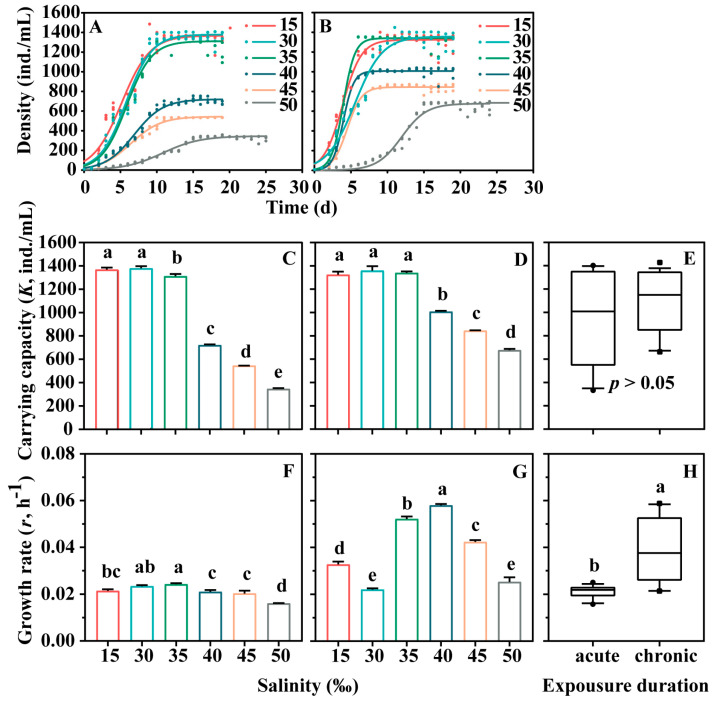
Population growth dynamics curves and the derived parameters of carrying capacity (*K*) and per capita growth rate (*r*) of *Euplotes vannus* exposed to different salinities. (**A**,**C**,**F**) Population growth dynamics, *K*, and *r* for acute stress (24 h) experiments, respectively. (**B**,**D**,**G**) Population growth dynamic parameters, *K*, and *r*, for chronic stress (336 h) experiments, respectively. In (**A**–**D**), data are presented as means ± S.E. (standard error); error bars represent the standard errors of the means (n = 3). Continuous lines in growth dynamics are the best fit to the data following logistic growth equation. Columns bearing the same letter are not significantly different as determined by the least significant difference (LSD) multiple range test when overall significant differences were detected (*p* = 0.05). In (**E**,**H**) Differences in population growth dynamic parameters between acute and chronic experimental groups were analyzed by *t*-test, where ‘a’ and ‘b’ represent significant differences.

The population growth dynamics of *E. vannus* displayed sigmoid responses to salinity changes and were characterized by distinct parameters (Figure 1). For the environmental capacity (*K*), the trend was consistent in both acute and chronic stress experiments. Specifically, no significant difference in *K* values was observed between the 15‰ and 30‰ treatment groups (*p* > 0.05), indicating that hypotonic stress had no significant effect on *K.* Conversely, the *K* value significantly decreased with increasing salinity from 30‰ to 50‰ (*p* < 0.05), suggesting a significant effect of hypertonic stress on *K.* Additionally, no significant difference in *K* values was detected between the ciliates from the acute and chronic experimental treatments (Figure 1E, *p* > 0.05). For the per capita growth rate (*r*), the trend differed between acute and chronic stress experiments. In the acute stress experiment, the *r* values decreased in *E. vannus* under both hypotonic and hypertonic stress, with a significant reduction observed as salinity increased from 30‰ to 50‰ (*p* < 0.05). In contrast, in the chronic stress experiments, *r* values increased significantly in *E. vannus* under both hypotonic (15‰) and hypertonic stress (from 35‰ to 45‰) (*p* < 0.05). Moreover, *r* values were significantly higher in ciliates from the chronic treatments compared to those from acute treatments (Figure 1H, *p* < 0.05). The results of the *r*-ANOVA revealed significant effects of salinity treatment, exposure duration, and treatment × duration interaction for *K* and *r* in *E. vannus* exposed to varying salinity levels for different durations (*p* < 0.05, Table 1).

### 3.2. Effects on Energy Storage Substance

**Figure 2:**
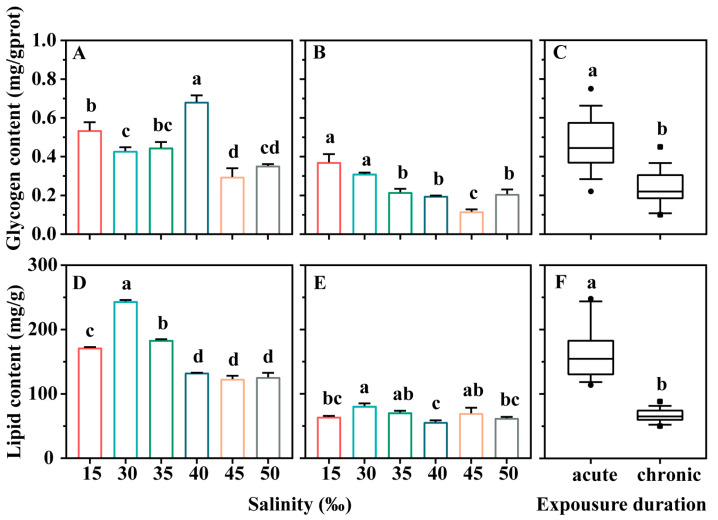
The contents of glycogen and lipid in *Euplotes vannus* exposed to different salinities. (**A**,**B**) Glycogen contents of *E. vannus* exposed to acute and chronic stress experiments, respectively. (**D**,**E**) Lipid contents for the acute and chronic stress experiments, respectively. In (**A**–**D**), data are presented as means ± S.E. (standard error); error bars represent the standard errors of the means (n = 3). Columns bearing the same superscript letter are not significantly different as determined by LSD multiple range test when overall significant differences were detected (*p* = 0.05). In (**C**,**F**), differences in contents of glycogen and lipid between the acute and chronic experimental groups were analyzed by *t*-test, where ‘a’ and ‘b’ represent significant differences.

Glycogen and lipid contents in *E. vannus* exhibited significant variations in response to salinity stress in acute and chronic stress experiments (Figure 2). In the acute stress experiments, hypotonic (15‰) and hypertonic stress (40‰) notably increased the glycogen content (Figure 2A, *p* < 0.05). However, glycogen content sharply declined when salinity exceeded 40‰ (Figure 2A, *p* < 0.05). In the chronic stress experiment, glycogen content increased with hypotonic stress (15‰) but decreased with hypertonic stress ranging from 35‰ to 50‰ (Figure 2B, *p* < 0.05). Regarding lipid content, both hypotonic (15‰) and hypertonic (35‰ to 50‰) significantly decreased lipid content in *E. vannus* during the acute stress experiment (Figure 2D, *p* < 0.05). A similar trend was observed in the chronic experiment, although the difference between the treatments diminished (Figure 2E, *p* < 0.05). The results of *r*-ANOVA indicated significant effects of salinity concentration and exposure duration on glycogen and lipid content in ciliates subjected to varying salinity concentrations for different durations in addition to the interaction between treatment and duration (*p* < 0.05, Table 1).

### 3.3. Effects on Energy Metabolism Enzyme Activity

**Figure 3:**
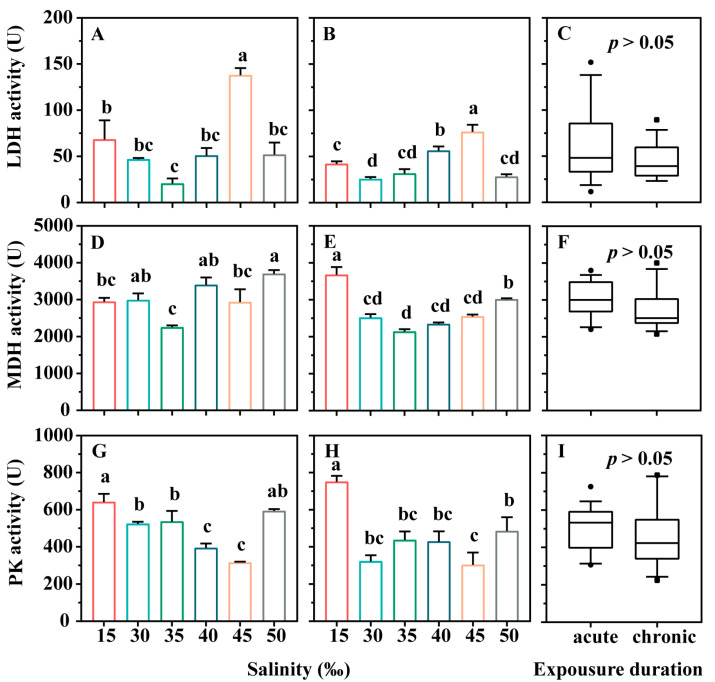
Enzyme activities of energy metabolism in *Euplotes vannus* exposed to different salinities. (**A**,**B**) Enzyme activities of lactate dehydrogenase (LDH) in *E. vannus* exposed to the acute (24 h) and chronic (336 h) stress experiments, respectively. (**D**,**E**) Malate dehydrogenase (MDH) for the acute and chronic stress experiments, respectively. (**G**,**H**) Pyruvate kinase (PK) for the acute and chronic stress experiments, respectively. In (**A**–**D**), data are presented as means ± S.E. (standard error); error bars represent the standard errors of the means (n = 3). Columns bearing the same superscript letter are not significantly different as determined by LSD multiple range test when overall significant differences were detected (*p* = 0.05). (**C**,**F**,**I**) The difference analysis of LDH, MDH, and PK activities between the acute and chronic experimental groups, respectively. Differences in LDH, MDH, and PK activities between the acute and chronic experimental groups were analyzed by *t*-test.

The activities of the energy metabolism enzymes were significantly influenced by the changes in salinity (Figure 3). For lactate dehydrogenase (LDH), hypotonic stress (15‰ salinity) had no significant effect in the acute stress experiments, while hypertonic stress significantly increased LDH activity as salinity increased to 45‰ (*p* < 0.05). However, ciliates treated with 50‰ salinity exhibited LDH activity comparable to that of the control group (30‰; *p* > 0.05, Figure 3A). In the chronic stress experiments, LDH activity displayed a trend similar to that observed in the acute experiments. Nevertheless, overall, the LDH activity of ciliates was higher in the acute experiments than in the chronic experiments (Figure 3C, *p* > 0.05). *r*-ANOVA results indicated that salinity treatment, exposure duration, and their interaction significantly affected LDH activity in *E. vannus* exposed to varying salinity levels over different time periods (*p* < 0.05, Table 1).

In the acute stress experiments, malate dehydrogenase (MDH) activity significantly decreased at 35‰ (Figure 3D, *p* < 0.05), with no significant difference observed between the other groups and the control group (Figure 3D, *p* > 0.05). In the chronic stress experiments, both hypertonic and hypotonic stress significantly increased MDH activity (Figure 3E, *p* < 0.05). However, no significant difference was found in MDH activity between ciliates from the acute and chronic experiments (Figure 3F, *p* > 0.05). The *r*-ANOVA results revealed that exposure duration had a significant effect on MDH activity in *E. vannus*, apart from the interaction between treatment and duration (*p* < 0.05, Table 1).

For the pyruvate kinase (PK) activity, hypotonic stress (15‰) significantly increased the PK activity in ciliates in the acute stress experiment (Figure 3G, *p* < 0.05), while hypertonic stress (40‰ and 45‰) presented opposite effects (Figure 3G, *p* < 0.05). In the chronic stress experiment, hypotonic stress (15‰) continued to enhanced PK activity, whereas hypertonic stress (35‰, 40‰, 45‰, and 50‰) had no significant effect (Figure 3H, *p* > 0.05). The results of *r*-ANOVA showed that both salinity and stress duration significant affected PK activity in *E. vannus*, with no significant interaction between salinity and duration (Table 1).

### 3.4. Gene Expression Levels of Enzymes Related to Energy Metabolism

**Figure 4:**
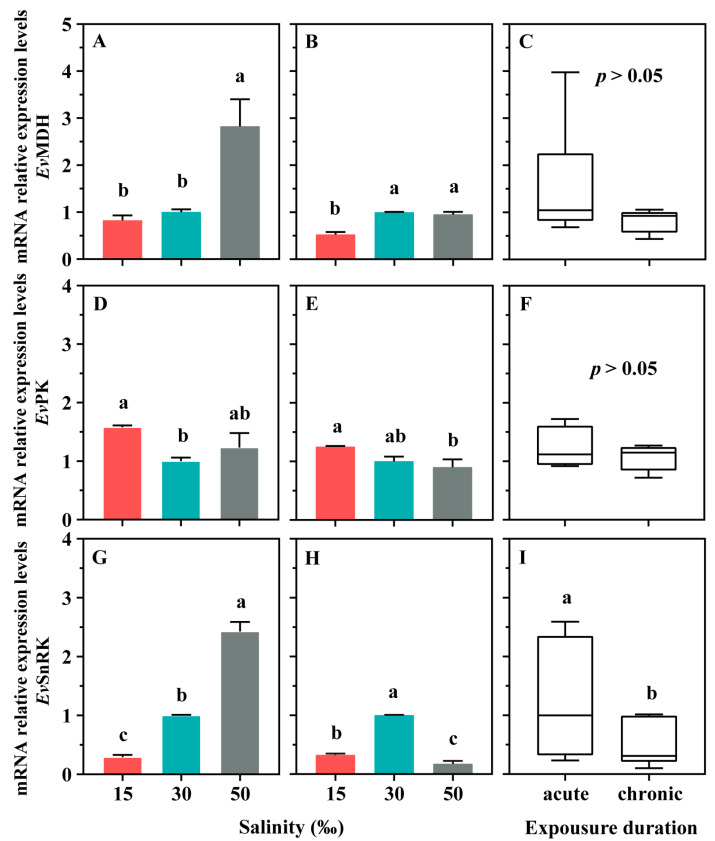
Effects of salinity exposure stress on the mRNA expression levels of malate dehydrogenase (MDH), pyruvate kinase (PK), and snf1-related protein kinase (SnRK) in the *Euplotes vannus*. (**A**,**D**,**G**) *Ev*MDH, *Ev*PK, and *Ev*SnRK for acute stress experiments, respectively. (**B**,**E**,**H**) *Ev*MDH, *Ev*PK, and *Ev*SnRK for chronic stress experiments, respectively. In (**A**,**B**,**D**,**E**,**G**,**H**), data are presented as means ± S.E. (standard error of the mean); error bars represent the standard errors of the means (n = 3). Columns bearing the same superscript letter are not significantly different as determined by LSD multiple range test when overall significant differences were detected (*p* = 0.05). (**C**,**F**,**I**) The difference analysis of *Ev*MDH, *Ev*PK, and *Ev*SnRK expression levels between the acute and chronic experimental groups, respectively. Differences in *Ev*MDH, *Ev*PK, and *Ev*SnRK expression levels between the acute and chronic experimental groups were analyzed by *t*-test, where ‘a’ and ‘b’ represent significant differences.

The results of the qRT-PCR indicated that salinity stress significantly impacted the relative mRNA expression levels of genes associated with energy metabolism (Figure 4). Specifically, hypertonic stress (50‰) significantly increased the expression of *Ev*MDH in the acute stress experiment (Figure 4A, *p* < 0.05), whereas hypotonic stress (15‰) had no significant effect (Figure 4A, *p* > 0.05). However, in the chronic stress experiment, the effects of hypertonic and hypotonic stress on *Ev*MDH gene expression exhibited an opposite trend (Figure 4B). For *Ev*PK, hypotonic stress (15‰) significantly increased gene expression in the acute experiment (Figure 4D, *p* < 0.05). In the chronic stress experiment, *Ev*PK gene expression was significantly higher under hypotonic (15‰) stress than that under hypertonic (50‰) stress in ciliates (Figure 4E, *p* < 0.05), but neither was significantly different from the control (Figure 4E, *p* > 0.05). For *Ev*SnRK, hypotonic stress (15‰) significantly decreased gene expression in the acute experiment, whereas hypertonic stress exerted the opposite effect (Figure 4G, *p* < 0.05). In the chronic stress experiment, both hypotonic stress (15‰) and hypertonic stress (50‰) reduced the expression levels of *Ev*SnRK (Figure 4H, *p* < 0.05).

### 3.5. Correlations between Growth Dynamic and Energy Metabolism Parameters

Correlations between growth dynamic parameters and energy metabolism variables were examined according to the durations of exposure to salinity concentrations (Table 2). In acute stress experiments, *r* exhibited significant positive correlations with lipid content and negative correlations with the mRNA expression of *Ev*MDH and *Ev*SnRK (*p* < 0.05). No significant correlations were found between glycogen content, lipid content, and enzyme activity, but a significant negative correlation was found between glycogen content, lipid content, and *Ev*MDH mRNA expression (*p* < 0.05). In the chronic stress experiments, *r* displayed a negative correlation only with *Ev*MDH mRNA expression. Glycogen content exhibited a significant negative correlation with LDH activity and a significant positive correlation with MDH and PK activities (*p* < 0.05), whereas no significant correlation was observed lipid content and enzyme activities. Furthermore, significant positive correlations were observed between glycogen content and *Ev*PK mRNA expression as well as between lipid content and *Ev*SnRK mRNA expression (*p* < 0.05). To further examine the effect of osmotic stress direction (hypotonic stress vs. hypertonic stress) on biological adaptability, we analyzed the trade-off between parameters under both hypotonic and hypertonic stress conditions (Table S4). These correlation analyses revealed that the growth strategy is closely linked to the trade-offs among different energy metabolism variables and exposure durations.

## 4. Discussion

Salinity, a critical environmental factor, significantly influences the functioning and stability of marine ecosystems. Extensive studies have been conducted on the biological responses to salinity changes and their ecological consequences in various marine organisms [7,8,9]. However, limited research has focused on the properties of salinity fluctuations. This is probably one of the key reasons for the disagreement among the different investigations. To clarify these controversies, it is crucial to select an appropriate model organism for comprehensive investigation under a unified experimental system [21]. Therefore, this study comprehensively examined the biological responses of ciliates to salinity changes such as fluctuation amplitude, shift direction, and exposure duration, encompassing population dynamics, energy content, and metabolic enzymes. As hypothesized in the introduction, our findings underscore the influence of salinity fluctuation properties on the adaptive strategies of the tested ciliates, and correspondingly, the response mechanisms of the energy metabolism vary in accordance with these properties.

### 4.1. Salinity Fluctuations on Population Growth Dynamics and Underlying Adaptive Strategies

Population growth is a reliable index for measuring environmental adaptability since it allows fitness to be understood as the integration of functions at subcellular levels with those of the whole organism [50]. Altered population growth can also indicate adaptive strategies of certain organisms to environmental changes, such as life-history strategies [51]. Furthermore, the *K*-strategy and *r*-strategy, extended by the parameters of population growth dynamics, have been widely used in ecological studies [22]. In this study, the population growth dynamic parameters of the *Euplotes vannus*, namely *K* and *r*, showed different change patterns influenced by the properties of salinity fluctuations. Specifically, *K* values in *E. vannus* challenged by hypertonic stress showed a downward trend in both acute and chronic tests. Although the hypotonic stress (15‰) had no significant effect on *K* in this study, the pre-experimental results indicated that the survival of the *E. vannus* drastically declined once the salinity was below 15‰ (unpublished data). According to the life-history theory [22,51], this is sufficient to rule out the possibility that the ciliates would adopt a *K*-strategy to cope with the salinity changes. Conversely, the variation trend of *r* was varied due to the properties of the salinity fluctuations. Under chronic stress, both hypotonic and hypertonic stress increased *r* values across salinity ranges of 15‰ to 40‰. This suggested that the *E. vannus* tested in our study adopted the *r*-strategy within the tolerate range of salinity stress. That is, *E. vannus* increased offspring numbers (expressed by *r*) but at the expense of the offspring quality (expressed by *K*) [52]. However, when the salinity exceeded a certain range (40‰), the *r* value showed a significant downward trend. Furthermore, in chronic stress tests, the relationship between *r* and salinity (especially in the hypertonic direction) conformed to the parabolic curve, which is the classic reaction norm between adaptive plasticity and environmental variables [53,54]. This indicated that the tested *E. vannus* gradually decreased in fitness as salinity stress intensity increased and was even lost once the salinity exceeded their tolerance threshold [53]. Similar results were also observed in *E. vannus* exposed to a gradient concentration of the phenol solution [25]. During acute stress tests, both hypotonic and hypertonic challenges reduced the *r* values, indicating that the tested *E. vannus* did not adopt the *r*-strategy. The result seems difficult to interpret for the following reasons. Extrapolating from the results of the chronic experiments, the salinity tested in the acute stress tests should not exceed the tolerance threshold of *E. vannus*. However, the specific adaptive strategies cannot be distinguished from the perspective of the dynamic relationship between *r* and *K*. This perplexity is most likely due to the trade-offs present in the life history of ciliates. As de Jong [55] stated, the organization of trade-offs is often hierarchical when considered from the point of view of resource allocation. Specifically, if the trade-offs between survival and reproduction are higher in the hierarchy than those among parentally influenced offspring traits, increasing the allocation of resources for reproduction correspondingly reduces or eliminates the trade-offs between offspring traits because of a surplus of resources dedicated to all offspring functions [22]. It can be inferred that in acute stress tests, the tested ciliates may allocate more energy to address urgent survival problems in near-term rather than long-term reproduction and therefore do not exhibit unique life-history strategies. In contrast, in chronic challenge tests, more energy is allocated to reproduction after long-term adaptation. Of course, it is necessary to further verify this inference in terms of the biological response at the lower organizational levels. As mentioned in the introduction, the perspective of energy metabolism might shed more light on the biological response mechanism of ciliates to salinity fluctuations.

### 4.2. Responding Mechanism to Salinity Fluctuations

Lipid and glycogen are the most important metabolic substances in invertebrate organisms [56,57]. They play a critical and multifunctional role in energy supply by generating ATP during metabolic processes [57]. In invertebrates, a high lipid content generally indicates good physiological conditions [58]. Furthermore, lipids serve as nutrient reserves when glucose supply is insufficient, for instance, during starvation [59] and during reproduction [60], indicating a central role in regulating life-history trade-offs [61]. Conversely, the level of glycogen signifies the energy content available for current activity [61]. Short-term energy costs due to physiological activities are more likely to affect glycogen content, while long-term physiological effects, such as those associated with a reallocation of energy, may affect lipid reserves [58]. In the acute stress experiments, both hypotonic and hypertonic stress enhanced the glycogen content when salinity varied between 15‰ and 40‰. It is suggested that the physiological activity of the tested *E. vannus* was enhanced within the range of salinity stress strengths that they can withstand. However, when salinity exceeded 40‰, glycogen content exhibited a significant decreasing trend, indicating that the salinity stress surpassed the self-regulation threshold of the tested *E. vannus* [54,62], resulting in decreased physiological activity. Obviously, the pattern of changes in glycogen content with salinity also fits well with the reaction norm between adaptive plasticity and environmental variables [53]. Accordingly, in chronic stress experiments, decreased glycogen content with salinity implies reduced physiological activity. Remarkably, the mean glycogen level was significantly higher in acute than in chronic experiments, potentially indicating higher physiological activity in acute stress tests [58]. Similar results were observed in the blue-spotted mudskippers (*Boleophthalmus pectinirostris*), where hypotonic salinity treatment increased their blood glucose concentration and eventually led to a decrease in hepatic glycogen content [63]. And it is possible to allocate more energy to enhance physiological activities to combat stress for survival [61,64]. Regarding lipid content, salinity stress (whether hypotonic or hypertonic) significantly reduced lipid levels of the tested *E. vannus*, and these effects are positively correlated with the duration and intensity of stress. Although the effects of salinity stress on lipid storage and utilization in marine animals are poorly reported, a study on turbot (*Scophthalmus maximus*) demonstrated that hypotonic stress causes lipid metabolism disorders [31]. This confirms that both the duration and intensity of stress can significantly reduce the physiological condition of the tested organism [58]. Besides dietary uptake, glucose can be provided by the mobilization of glycogen stores or gluconeogenesis [65]. Two key biochemical processes closely related to glucose metabolism are glycolysis and the TCA cycle [66,67]. Thus, biological responses related to energy metabolism have been widely used as valuable biomarkers for the evaluation of ecological impacts due to environmental changes [68]. For instance, pyruvate kinase (PK) is a key enzyme controlling metabolic flux and ATP production, as it catalyzes the irreversible conversion of ADP and phosphoenolpyruvate to ATP and pyruvic acid in the last step of glycolysis [69]. Moreover, lactate dehydrogenase (LDH) participates in the regulation of anaerobic glycolysis and gluconeogenesis [70]. Additionally, malate dehydrogenase (MDH) catalyzes the reaction between malate and NAD^+^, producing oxaloacetate and NADH and playing a crucial role in the tricarboxylic acid cycle [71]. In this study, both hypotonic and hypertonic challenges tended to improve the activity of these three metabolic enzymes, and salinity and duration had significant interactive effects on enzyme activity. This further confirms that the enhanced physiological activity of ciliates under salinity stress could cause corresponding changes in energy metabolism in the organism.

Compared to metabolic enzyme activity, their gene expression levels are generally more sensitive in response to environmental stress. Furthermore, as a useful supplement for enzyme activity, gene expression profiling based on mRNA has been used to identify ideal biomarkers of potential environmental pressure [72]. Among the three genes determined in this study, SnRK 1 belongs to the protein kinase family and is a homolog of mammalian AMPK [73]. AMPK has been confirmed to promote lipolysis and glycolysis through the activation of key enzymes such as lactate dehydrogenase (LDH), succinate dehydrogenase (SDH), pyruvate kinase (PK), and hexokinase (HK) [74]. Furthermore, it has also been demonstrated that salinity stress inhibits fat synthesis and promotes fat oxidation in the turtle (*Trachemys scripta elegans*) liver, and this effect is regulated by the AMPK activation pathway [75]. Previous studies have shown the great potential of the gene expression levels of SnRK 1 (*Ev*SnRK) as a sensitive indicator in response to salt stress [76]. Similarly, gene expression of both pyruvate kinase (*Ev*PK) and malate dehydrogenase (*Ev*MDH) in response to environmental stress has been reported, for example, *Ev*PK in white-leg shrimp (*Litopenaeus vannamei*) to short-term hypoxia [77] and *Ev*MDH in soybean in response to salt stress [78]. In this study, the expression level of the three genes was significantly affected by salinity and stress duration, and interactive effects between the two were significant. This indicates that the response mechanism of the tested ciliates to salinity stress changes over salinity fluctuation properties. This requires a comprehensive disclosure of the biological response mechanism under the influence of salinity fluctuation properties by technologies such as genomics.

### 4.3. Trade-Off in Life-History Strategy Influenced by Salinity Fluctuations

Life-history strategies are strongly influenced by trade-offs between traits that strongly affect fitness [22,79]. The parameters determined in this experiment largely represent traits of the tested ciliates and are closely correlated with fitness. Specifically, metabolic enzyme activity and their gene expression reflect the current physiological activities of the organism and are important indicators of the body against environmental stress for survival [63,68,80]. In this study, metabolic enzyme activities and their gene expression levels in acute stress tests were generally higher than in chronic tests, indicating high physiological activity of ciliates under acute salt stress. As mentioned above, glycogen content implies direct energy reserves available for current physiological activities [61]. Moreover, lipids represent a long-term accumulation of excess energy, reflecting the physiological state of the body and serving as a potential energy source for offspring through reproduction [58]. In the acute stress tests, *r* was positively correlated with parameters related to energy reserves, especially lipid content, and conversely, *r* was negatively related to parameters associated with metabolic enzyme activity, including enzyme activity and gene expression. Notably, both *r* and lipid content decreased with increasing salinity stress intensity. Consequently, the increased physiological activity of tested ciliates for survival comes at the expense of reduced reproduction and energy storage under acute stress conditions. That is, in acute stress tests, a trade-off between survival and reproduction exists, where survival absolutely dominates. For the chronic stress experiment, as *r* increased due to salinity stress, it was negatively correlated with energy reserve, metabolic enzyme activity, and related gene expression levels. This suggests that the weight of reproduction increases in the reproduction vs. survival trade-off but at the cost of reduced physiological activity and diminished energy reserves. Similarly, the trade-off between survival and immediate reproduction was observed in young adult female mosquitoes (*Anopheles gambiae*) and was modulated based on resource availability and gonotrophic state [81]. In summary, the adaptive strategy of ciliates under salinity stress is regulated by the trade-off between reproduction and survival, with the outcome being influenced by salinity fluctuation properties, such as stress duration and intensity.

## 5. Conclusions

This study is the first to explore the adaptive strategies and potential response mechanisms of ciliated protozoa to salinity changes, particularly highlighting the effects of salinity fluctuation properties, including exposure duration (acute and chronic), shift direction (hypotonic and hypertonic), and concentration (from 15‰ to 50‰). According to the population dynamics parameters, the tested ciliates mainly adopted the *r*-strategy but were affected by the properties of the salinity fluctuations. Specifically, in the acute stress tests, ciliates did not show distinguished adaptive strategies because both hypertonic and hypotonic stresses led to decreases in *r* and *K* with increasing salinity stress intensity. In the chronic stress tests, ciliates adopted the *r*-strategy within the range of salinity stress strengths that they can tolerate. Regarding the biological response mechanisms, from energy storage content to gene expression levels, the biological response patterns were significantly distinguished by the properties of salinity fluctuations. Firstly, under acute salinity stress, both hypotonic and hypertonic salt intensity significantly increased glycogen content. When the stress intensity exceeded a certain threshold (40‰), the glycogen level decreased significantly. However, both hypotonic and hypertonic salt intensity significantly reduced glycogen content in ciliates under chronic salinity stress. For lipid levels, whether hypotonic or hypertonic, the salinity stress significantly reduced lipid levels of the tested *E. vannus* under both acute and chronic stress, and these effects were negatively correlated with the duration and intensity of stress. Secondly, both hypotonic and hypertonic stresses tended to improve the metabolic enzyme activities, and salinity and duration had significant interactive effects on these enzyme activities. Finally, the expression levels of these metabolic enzymes varied with salinity but showed no distinctive pattern. Thus, further studies utilizing genomic approaches are necessary to reveal deeper biological response mechanisms, particularly the distinction and connection between responses to hyper- and hypoosmotic stresses. Notably, the correlation between different biological levels demonstrated the trade-off between survival and reproduction. Specifically, under acute salinity stress, the trade-off occurred between survival and reproduction, with survival being absolutely dominant. However, under chronic salinity stress, the weight of reproduction increased in the reproduction versus survival trade-off, albeit at the cost of reduced physiological activity and energy reserves. In summary, this study enhances our understanding of adaptive strategies to salinity changes, elucidating the response mechanisms of ciliated protozoa to salinity while highlighting the fluctuation property. This provides a foundation for a reasonable assessment of the ecological effects of salinity changes in marine microecosystems.

## Figures and Tables

**Table 1:** Results of *r*-ANOVA for responses of ciliate variables following exposure to salinity.

Variable	Source of Variation	df	*F*-Values	*p*-Values
*K*	Time	1	330.813	0.000
	Salinity	5	674.154	0.000
	Time × Salinity	5	79.782	0.000
*r*	Time	1	827.119	0.000
	Salinity	5	176.471	0.000
	Time × Salinity	5	85.741	0.000
LDH	Time	1	13.721	0.003
	Salinity	5	25.917	0.000
	Time × Salinity	5	4.069	0.022
MDH	Time	1	9.685	0.011
	Salinity	5	0.588	0.710
	Time × Salinity	5	6.646	0.006
PK	Time	1	5.296	0.040
	Salinity	5	7.642	0.002
	Time × Salinity	5	3.012	0.055
Glycogen	Time	1	192.714	0.000
	Salinity	5	23.664	0.000
	Time × Salinity	5	12.163	0.000
Lipid	Time	1	3059.075	0.000
	Salinity	5	61.116	0.000
	Time × Salinity	5	89.660	0.000
*Ev*MDH mRNA expression	Time	1	11.518	0.015
	Salinity	2	16.000	0.004
	Time × Salinity	2	7.408	0.024
*Ev*PK mRNA expression	Time	1	4.845	0.070
	Salinity	2	6.616	0.030
	Time × Salinity	2	1.211	0.362
*Ev*SnRK mRNA expression	Time	1	247.404	0.000
	Salinity	2	75.237	0.000
	Time × Salinity	2	258.763	0.000

**Table 2:** Correlation analyses between parameters derived from logistic growth dynamics and energy metabolism variables using the Pearson correlation coefficient.

Factor	Acute Stress Experiment	Chronic Stress Experiment
*r* × Glycogen content	0.180 (−)	−0.448 (+)
*r* × lipid content	0.678 ^b^ (−)	−0.327 (+)
*r* × LDH	−0.297 (+)	0.461 (−)
*r* × MDH	−0.512 (+)	−0.440 (+)
*r* × PK	0.006 (−)	−0.166 (+)
*r* × *Ev*MDH mRNA	−0.802 ^a^ (+)	−0.921 ^b^ (+)
*r* × *Ev*PK mRNA	−0.116 (+)	0.377 (−)
*r* × *Ev*SnRK mRNA	−0.793 ^a^ (+)	−0.549 (+)
Glycogen content × LDH	−0.304 (+)	−0.514 ^a^ (+)
Glycogen content × MDH	0.002 (−)	0.590 ^a^ (−)
Glycogen content × PK	0.023 (−)	0.713 ^b^ (−)
Lipid content × LDH	−0.415 (+)	−0.234 (+)
Lipid content × MDH	−0.271 (+)	−0.270 (+)
Lipid content × PK	0.358 (−)	−0.143 (+)
Glycogen content × *Ev*MDH mRNA	−0.704 ^a^ (+)	−0.485 (+)
Glycogen content × *Ev*PK mRNA	0.466 (−)	0.698 ^b^ (−)
Glycogen content ×*Ev*SnRK mRNA	−0.846 ^b^ (+)	0.273 (−)
Lipid content × *Ev*MDH mRNA	−0.675 ^a^ (+)	0.420 (−)
Lipid content × *Ev*PK mRNA	−0.533 (+)	−0.269 (+)
Lipid content × *Ev*SnRK mRNA	−0.317 (+)	0.891 ^b^ (−)

Notes: + denotes tradeoff; − denotes no tradeoff. ^a^ Correlation is significant at the 0.05 level (2-tailed). ^b^ Correlation is significant at the 0.01 level (2-tailed).

## Data Availability

The data that support the findings of this study are available from the corresponding authors.

## Supplementary Materials

The following supporting information can be downloaded at: https://www.mdpi.com/article/10.3390/microorganisms12101957/s1, Text S1: Details of sample collection, total RNA extraction, gene cloning, cDNA synthesis, and real-time quantitative PCR; Table S1: Primer sequences for RACE-PCR amplification of genes; Table S2: Primer sequences for qRT-PCR amplification of genes; Table S3: qPCR system and qPCR amplification program; Table S4: Correlation analysis of growth dynamic parameters under hypotonic and hypertonic stress with energy metabolism variables using the Pearson’s correlation coefficient. References [75,82] are cited in the Supplementary Materials.
