# Supplementary material for: Adaptive Strategies and Underlying Response Mechanisms of Ciliates to Salinity Change with Note on Fluctuation Properties
Source: Microorganisms. 2024 Sep 27;12(10):1957. doi: 10.3390/microorganisms12101957 (PMC11509147; doi:10.3390/microorganisms12101957)
Supplement: Supplementary file 1 [file microorganisms-12-01957-s001.zip › microorganisms-3227663-supplementary.pdf]

Supplementary Materials

Text S1. Details of sample collection, total RNA extraction, gene cloning, cDNA synthesis, real-time quantitative PCR.

Euplotes vannus was cultured under constant temperature conditions until it reached the logarithmic growth phase. The culture was then rinsed with sterilized seawater and prepared as suspensions with varying salt concentrations. In acute exposure experiments, the ciliate suspensions were cultured in a constant-temperature incubator for 24 hours. For chronic exposure experiments, the ciliate suspensions were cultured under the same conditions for 14 days. Each experimental group was tested in triplicate. Subsequently, the culture was filtered and enriched, and then subjected to a 12-hour starvation period before total RNA extraction. To analyze the influence of hypotonic and hypertonic stress on the energy metabolism genes of *Euplotes vannus* across different exposure durations, we cloned three genes: *Ev*MDH, *Ev*PK, and *Ev*SnRK. The methods for total RNA and gene expression analyses are detailed in [82]. The integrity of RNA was evaluated by 1% agarose gel electrophoresis (Transgen Biotech, Beijing, China), focusing on the 18S and 28S rRNA bands, and its concentration was measured using a Nano-Drop 2000 spectrophotometer (Thermo Fisher, USA) to determine the OD260/OD280 ratio.

The method for obtaining the full-length cDNAs of the genes is detailed by Huang et al. [75]. The PCR was performed using the SMART RACE cDNA 5′/3′ Kit (TaKaRa, Japan) with gene-specific primers (listed in Table S1). The specific primer sequences were synthesized by Tianyihuiyuan Gene Technology Co., Ltd. (Guangzhou, China). The RACE PCR was conducted as follows: 5 cycles at 94 °C for 30 sec, 72 °C for 3 min; 5 cycles at 94 °C for 30 sec, 70 °C for 30 sec, and 72 °C for 3 min; and 5 cycles at 94 °C for 30 sec, 68 °C for 30 sec, and 72 °C for 3 min. Nested PCR was performed using the RACE kit primers NUP, 3’-RACE2, and 5’-RACE2. The target products were gel-extracted and purified following the instructions of the Universal DNA Purification Kit (TIANGEN, Beijing, China). Subsequently, the target genes were ligated into the pEASY-T1 vector according to the pEASY-T1 Cloning Kit and transformed into *Escherichia coli* DH5α competent cells. Antibiotic selection using LB agar plates containing ampicillin was performed to screen for ampicillin-resistant colonies (containing recombinant plasmids). Resistant colonies were selected and grown overnight in liquid LB medium containing ampicillin. Liquid cultures with correct PCR product size were selected and submitted to Guangzhou Tianyihuiyuan Gene Technology Co., Ltd. for sequencing and identification.

According to the obtained sequences, *Ev*MDH, *Ev*PK, and *Ev*SnRK were obtained for gene expression analysis through qPCR. The cDNA was synthesized using Hiscript® III RT

SuperMix (+gDNA wiper) (Vazyme, Nanjing, China). Real-time PCR was performed with ChamQ SYBR qPCR Master Mix ((LOW ROX Premixed) (Vazyme, Nanjing, China) on QuantStudio® 5 (Applied Biosystems, USA). The α-tubulin gene of *E. vannus* (GenBank accession no. Z11769) and ribosomal protein s6 (unpublished transcriptome database) was used as internal control genes, based on our preliminary study, which showed that the amplicon efficiencies of the target and reference genes were approximately equal. The detailed description of the primers is provided in Table S2. The qPCR system and qPCR amplification program are detailed in Table S3. A melting curve analysis of the amplified product is performed at the end of each reaction to determine the formation of a specific product.

Table S1. Primer sequences for RACE-PCR amplification of genes.

**Primer name**	**Nucleotide sequence (5' to 3')**
10×UPM	CTAATACGACTCACTATAGGGCAAGCAGTGGTATCAAC GCAGAGT
NUP (10μM)	CTAATACGACTCACTATAGGGC
GSP-MDH-5' RACE1	GATTACGCCAAGCTTCCATTCCAGGACCTCTTGGCTTC G
NGSP-MDH-3' RACE1	GATTACGCCAAGCTTGGTGCGAAGCCAAGAGGTCCTG GAAT
GSP-MDH-3' RACE2	GATTACGCCAAGCTTTCTGAGCTACATGCCCTGAAGCA GGA
NGSP-MDH-5' RACE2	GATTACGCCAAGCTTCCTGCTTCAGGGCATGTAGCTCA G
GSP-PK-5' RACE1	GATTACGCCAAGCTTCGGGCATTTGTGTAGCCGTGATA ACTGG
NGSP-PK-3' RACE1	GATTACGCCAAGCTTGGTGCGAAGCCAAGAGGTCCTG GAAT
GSP-PK-3' RACE2	GATTACGCCAAGCTTCACGGCTACACAAATGCCCGAGT CAATG
NGSP-PK-5' RACE2	GATTACGCCAAGCTTCTGGAGGGATTTCCATACCAAGG TCTCC
GSP-SnRK-5' RACE1	GATTACGCCAAGCTTCGGGCATTTGTGTAGCCGTGATA ACTGG
NGSP-SnRK-3' RACE1	GATTACGCCAAGCTTGGATCTGATTGTGTCATGCTTTCT GGTG
GSP-SnRK-3' RACE2	GATTACGCCAAGCTTCACGGCTACACAAATGCCCGAGT CAATG
NGSP-SnRK-5' RACE2	GATTACGCCAAGCTTCTGGAGGGATTTCCATACCAAGG TCTCC

Table S2. Primer sequences for qRT-PCR amplification of genes.

Primer name	(5´ to 3´) nucleotide sequence	fragment size (bp)
MDH-RT-F	TTGAACGCTCCAAGTATCCC	208
MDH-RT-R	TCTCCACGAATCCCTCAGTT	
PK-RT-F	AACCAGGAGGGACTCAACAAC	188
PK-RT-R	ATCATTGACTCGGGCATTTGT	
SnRK-RT-F	GCTCATCCATGCTATTGCCTTTA	206
SnRK-RT-R	CAAGATTGGAGATTTTGGAGTTA	
Tubulin-RT-F	GCACACTGATGTCGCTGTTATGCTTGA	122
Tubulin-RT-R	AGGAGATAACCTGGGCGATGAGTC	
rps6-F	GGAAACGACAAGCAAGGA	102
rps6-R	AGTGAGAGCGATTACGGC	

Table S3. qPCR system and qPCR amplification program.

1. qPCR system

**Component**	**Volume of addition (20 µl reaction)**
2 × ChamQ SYBR qPCR Master Mix (LOW ROX Premixed)	10.0 µl
Primer-F (10 µM)	0.4 µl
Primer-R (10 µM)	0.4 µl
cDNA	2 µl (100 ng)
ddH_2O	7.2 µl

Mix include dNTP, Mg^{2+}, Champagne Taq DNA Polymerase, SYBR Green I, ROX Reference Dye 2, etc.

2. qPCR amplification program

**Stage 1**	**Initial denaturation**	**Reps: 1**	**95℃**	**30 sec**
**Stage 2**	Circular reaction	Reps: 40	95℃	10 sec
			60℃	30 sec
**Stage 3**	Dissociation curve	Reps: 1	95℃	15 sec
			60℃	30 sec
			95℃	10 sec

Table S4. Correlation analysis of growth dynamic parameters under hypotonic and hypertonic stress with energy metabolism variables using the Pearson's correlation coefficient.

**Variable**	**Hypotonic stress (15-30 ‰)**		**Hypertonic stress (30-50 ‰)**	
	A	C	A	C
r × Glycogen	**-0.971**[b] (+)	0.622 (–)	0.205 (–)	-0.475 (+)
r × Lipids	0.788 (–)	-0.776 (+)	**0.682**[b] (–)	-0.378 (+)
r × LDH	-0.804 (+)	0.811 (–)	-0.273 (+)	0.471 (–)
r × MDH	0.102 (–)	**0.964**[b] (–)	-0.528 (+)	**-0.621**[a] (+)
r × PK	-0.324 (+)	**0.942**[a] (–)	-0.064 (+)	0.190 (–)
r × *Ev*MDH mRNA	0.290 (–)	**-0.943**[b] (+)	**-0.816**[a] (+)	**-0.937**[b] (+)
r × *Ev*PK mRNA	-0.767 (+)	0.784 (–)	-0.424 (+)	**-0.816**[a] (+)
r × *Ev*SnRK mRNA	0.805 (–)	**-0.966**[b] (+)	**-0.972**[b] (+)	-0.612 (+)
Glycogen × LDH	**0.858**[a] (–)	0.540 (–)	-0.416 (+)	**-0.728**[b] (+)
Glycogen × MDH	-0.017 (+)	0.681 (–)	0.021 (–)	-0.082 (+)
Glycogen × PK	0.299 (–)	0.599 (–)	-0.099 (+)	0.250 (–)
Lipids × LDH	-0.486 (+)	**-0.950**[b] (+)	-0.438 (+)	-0.237 (+)
Lipids × MDH	0.080 (–)	**-0.824**[a] (+)	-0.270 (+)	-0.156 (+)
Lipids × PK	-0.778 (+)	-0.796 (+)	0.377 (–)	-0.038 (+)
Glycogen × *Ev*MDH mRNA	-0.396 (+)	-0.401 (+)	**-0.847**[a] (+)	0.603 (–)
Glycogen × *Ev*PK mRNA	0.771 (–)	0.493 (–)	-0.298 (+)	0.558 (–)
Glycogen ×*Ev*SnRK mRNA	-0.807 (+)	-0.624 (+)	**-0.815**[a] (+)	**0.934**[b] (–)
Lipids × *Ev*MDH mRNA	0.586 (–)	**0.878**[a] (–)	**-0.865**[a] (+)	0.199 (–)
Lipids × *Ev*PK mRNA	**-0.983**[b] (+)	**-0.882**[a] (+)	-0.319 (+)	0.029 (–)
Lipids × *Ev*SnRK mRNA	**0.994**[b] (–)	**0.865**[a] (–)	**-0.956**[b] (+)	**0.893**[a] (–)

Notes: +, denotes tradeoff; –, denotes no tradeoff. Bold entries highlight the significant correlation. A: acute stress experiments, C: chronic stress experiments.

[a] Correlation is significant at the 0.05 level (2-tailed).

[b] Correlation is significant at the 0.01 level (2-tailed).
